# The Book World of Medicine and Science

**Published:** 1902-04-19

**Authors:** 


### The Book World of Medicine and Science.

The Medical Annual: A Year-book of Treatment and Practitioner's Index, 1902. (Bristol: John Wright and Co. Price 7s. 6d. net.)

It is always with pleasure that we welcome the appearance of this admirable summary of the year's progress, and the volume now before us serves well to show how great that progress really is. The subjects dealt with are well selected, the articles are well written, the illustrations are freely introduced, and the whole book is well up to date. Among the articles which may be specially referred to are one by Dr. Howard Gladstone describing aseptic surgery as practised in New York; a very complete and useful article on the clinical examination of the blood by Dr. Kelynack, and one on arsenical poisoning by the same author; one on disorders of metabolism, written by Dr. Bertram Abrahams, in which the uses of the various internal secretions are discussed; one on high frequency currents, which will serve to supply at least a general outline of the subject; and a very practical article on vision, in which Mr. A. St. Clair Buxton describes the methods of examining the eye for errors of refraction and accommodation. This article is well illustrated by full-sized test types, etc., and is likely to prove useful to those who are not what the author calls "proficient refractionists." Among the other contents of this very useful annual we note a series of legal decisions affecting the public health and the medical profession by Dr. J. E. Cooney of the Middle Temple.

Burdett's Hospitals and Charities, 1902. Being the Year-book of Philanthropy and the Hospital Annual. By Sir Henry Burdett, K.C.B. (Scientific Press. Price 5s.)

The continual advance in size and wealth of detail presented by "Burdett's Hospitals and Charities" year by year is evidence of the continued growth throughout the Empire of institutions dedicated to the relief of sickness and distress, whether supported by voluntary contributions or otherwise. Thirteen years ago, when the "Hospital Annual" was issued for the first time, many of the salient features of the hospital system were either altogether lacking, or were in their rudimentary stages. The great workhouse infirmary training schools which now offer advantages to the probationer, scarcely inferior to what may be found in the best general hospitals, might then be counted on the fingers of one hand. The Metropolitan Asylums Board have doubled the number of their hospitals, and far more than doubled their accommodation, while provincial isolation hospitals have greatly increased in number. A general tendency to growth is observable in every direction, and the careful observer may detect numberless instances of institutions now among the best of their kind, which thirteen years ago were listlessly managed on a system of *laisser faire*, to the discomfort of all concerned. It would be an interesting but we fear a difficult problem to ascertain the number of additional beds which have been added during this period to the hospital accommodation of the kingdom. The number is not probably out of the ratio of the increase of the population, and unless a check should occur in the growth of the population the increased demand for hospital accommodation will certainly be maintained. The book before us, however, has been doubled in size during the last thirteen years, not merely by additions made to the number of the institutions with which it deals. A great deal of the information contained in it has evidently been obtained only by patient research. Here we have over 100 closely-printed pages giving information relating to Colonial hospitals and British hospitals abroad. Anyone who has conscientiously tried to get information about such matters knows the extraordinary delays and hindrances which block the way, and can survey these concise paragraphs with the admiration of a connoisseur. W find new features added every year. The list of military and naval hospitals is a most important addition in this edition. The chapter on the comparative cost of the in-patient in different parts of the Empire is extremely interesting, and we look to see it further developed as time goes on. The number of Poor Law Infirmaries dealt with is again increased, several additional unions having established training schools and secured a place in the list. While fully appreciating the difficulty of dealing with the sick wards of all provincial workhouses, we would emphasise our conviction that nothing is so likely to bring improvement into these places as the publication in this volume of a list of them, showing briefly the number of beds and the number of nurses and attendants attached to each. We confidently commend the book to the attention of all who are interested in hospitals, whether as subscribers or officials, to be used not merely for purposes of reference, but for careful study. If not purely optimistic in tone, for it treats of suffering in all its phases, it opens up a vista of organised national effort and cosmopolitan sympathy which might restore hope in human nature even to those most inclined to cynicism about their fellow-men.

